# A Novel Cr_2_O_3_/MnO_2-x_ Electrode for Lithium-Oxygen Batteries with Low Charge Voltage and High Energy Efficiency

**DOI:** 10.3389/fchem.2021.646218

**Published:** 2021-02-01

**Authors:** Zhaohuan Wei, Zhiyuan Zhang, Yaqi Ren, Hong Zhao

**Affiliations:** ^1^ School of Physics, University of Electronic Science and Technology of China, Chengdu, China; ^2^ School of Materials and Environmental Engineering, Chengdu Technological University, Chengdu, China; ^3^ School of Materials Science and Energy Engineering, Foshan University, Foshan, China

**Keywords:** lithium-oxygen battery, MnO_2-x_, Cr_2_O_3_, Energy efficiency, charge voltage

## Abstract

A high energy efficiency, low charging voltage cathode is of great significance for the development of non-aqueous lithium-oxygen batteries. Non-stoichiometric manganese dioxide (MnO_2-x_) and chromium trioxide (Cr_2_O_3_) are known to have good catalytic activities for the discharging and charging processes, respectively. In this work, we prepared a cathode based on Cr_2_O_3_ decorated MnO_2-x_ nanosheets via a simple anodic electrodeposition-electrostatic adsorption-calcination process. This combined fabrication process allowed the simultaneous introduction of abundant oxygen vacancies and trivalent manganese into the MnO_2-x_ nanosheets, with a uniform load of a small amount of Cr_2_O_3_ on the surface of the MnO_2-x_ nanosheets. Therefore, the Cr_2_O_3_/MnO_2-x_ electrode exhibited a high catalytic effect for both discharging and charging, while providing high energy efficiency and low charge voltage. Experimental results show that the as-prepared Cr_2_O_3_/MnO_2-x_ cathode could provide a specific capacity of 6,779 mA·h·g^−1^ with a terminal charge voltage of 3.84 V, and energy efficiency of 78%, at a current density of 200 mA·g^−1^. The Cr_2_O_3_/MnO_2-x_ electrode also showed good rate capability and cycle stability. All the results suggest that the as-prepared Cr_2_O_3_/MnO_2-x_ nanosheet electrode has great prospects in non-aqueous lithium-oxygen batteries.

## Introduction

Non-aqueous lithium-oxygen batteries have been considered as a promising power source for portable devices and electric vehicles due to their high energy density (1.14 × 10^4^ Wh kg^−1^) (Wang et al., 2019; Kwak et al., 2020). However, several issues, such as high charge voltage, low energy efficiency, and short cycle life, need to be addressed before this technology is commercially viable (Eftekhari and Ramanujam, 2017; Wang et al., 2018). The abovementioned issues can be mainly attributed to the sluggish reaction dynamics of both, the cathode oxygen reduction reaction (ORR) and the oxygen evolution reaction (OER) (Li and Chen, 2017; Lim et al., 2017; Huang and Peng, 2019). Typically, during discharge, the lithium metal is oxidized to lithium ion at the negative electrode, and the oxygen is reduced to form an insoluble product, i.e., lithium peroxide (Li_2_O_2_), at the positive electrode. During charging, the discharge product, Li_2_O_2_, is converted into lithium ions and oxygen by electrochemical decomposition at the positive electrode, and the lithium ions are reduced and deposited at the negative electrode (Liu et al., 2019; Shu et al., 2019). The reaction at the negative electrode is known to be more reversible, with a faster reaction rate, while the reaction at the positive electrode suffers from poor reversibility and low reaction rate (Lim et al., 2017; Shu et al., 2019). Therefore, the ORR/OER reaction resistance dominates the total resistance. Many catalytic materials have been prepared to promote the electrochemical reaction and improve battery performance, such as carbon-based materials (Wu et al., 2020b; Li et al., 2020c; Falinski et al., 2020; Ren et al., 2020), noble metal/metal oxides (Li et al., 2020a; Nam et al., 2020; Zhu et al., 2020), and transition metal oxides (Wu et al., 2020a; Cai et al., 2020; Song et al., 2020).

Manganese dioxide (MnO_2_) based materials have received great attention as cathodes for lithium-oxygen batteries, because of their good stability and excellent catalytic activity for oxygen reduction reactions (Bi et al., 2019; Cheng et al., 2019; Yao et al., 2019; Dai et al., 2020). Among MnO_2_-based materials, non-stoichiometric manganese dioxide (MnO_2-x_), which contains oxygen vacancies and trivalent manganese, can significantly increase the conductivity and enhance the adsorption of oxygen species on the electrode surface (Zhai et al., 2014; Chen et al., 2015; Liu et al., 2018). Several studies have been conducted which demonstrate the high discharge capacity of MnO_2-x_ (Song et al., 2013; Hu et al., 2014). However, its catalytic activity for oxygen evolution reaction, during the charging process, is still not satisfactory and needs to be further improved (Luo et al., 2018; Zhang et al., 2019).

Chromium trioxide (Cr_2_O_3_) has a unique catalytic effect on the charging process of lithium-oxygen batteries. Yao et al. first studied its charging performance and showed that chromium-based materials could promote the discharge product decomposition through a solid-activation process by the mixed valence states Cr^3+^/Cr^6+^ on the interface at the Li_2_O_2_/Cr_2_O_3_ interface (Yao et al., 2014). Since then, a number of works have been carried out, and the results proved the high OER catalytic ability of Cr_2_O_3_ in lithium-oxygen batteries (Gan et al., 2016; Zhang et al., 2016). Thus, it is highly beneficial to use Cr_2_O_3_ to improve the charge performance of the MnO_2-x_ cathode.

In this work, we prepared a cathode based on Cr_2_O_3_ decorated MnO_2-x_ nanosheets using a simple adsorption process and applied this novel cathode as a binder-free, non-carbon cathode for non-aqueous lithium-oxygen batteries. In this electrode, a small number of Cr_2_O_3_ particles are uniformly decorated on the MnO_2-x_ surface, providing the following three advantages: 1) the MnO_2-x_ nanosheets provide high surface area for ORR and deliver high discharge capacity; 2) Cr_2_O_3_ promotes the formation of discharge product to achieve low charging voltage; and 3) a low loading of evenly distributed Cr_2_O_3_ on the surface of MnO_2-x_ nanosheets can minimize the inhibition effect on the oxygen reduction process and catalyze the Li_2_O_2_ formation. These striking features enable the Cr_2_O_3_/MnO_2-x_ nanosheet electrode to achieve high discharge capacity, high energy efficiency, and low charge voltage.

## Experimental Section

### Electrode Preparation

The MnO_2_ nanosheet electrode was prepared with a simple electrodeposition method. Manganese acetate (C_4_H_6_MnO_4_·4H_2_O), chromium nitrate (Cr(NO_3_)_3_·9H_2_O), and sodium sulfate (Na_2_SO_4_) were purchased from Aladdin, China. Stainless steel (SS) felt substrates were first cleaned in an H_2_SO_4_ solution, rinsed in distilled (DI) water, and air-dried at 60°C. The SS felt substrate was then immersed in a solution containing 0.1 M Na_2_SO_4_ and C_4_H_6_MnO_4_ with an anodic current density of 0.25 mA·cm^−2^ to obtain an electrodeposited layer of MnO_2_ nanosheets. Then, the as-obtained deposition was rinsed in DI water, dried at 90°C for 12 h, and was used as the MnO_2_ electrode. The loading of MnO_2_ was controlled to achieve 0.5 mg·cm^−2^ by adjusting the electrodepositing time and weighing.

After electrodeposition, the as-prepared MnO_2_ electrode was immersed in a 0.1 M (NH_4_)_2_CrO_4_ solution for 6 h to achieve the full adsorption of Cr ions. After the above treatment, the electrode was taken out of the solution, and the remaining liquid on the electrode surface was wiped dry by filter paper. The electrode was dried at 90°C, and calcined at 350 °C for 3 h, under N_2_ atmosphere. The resulting electrode was named Cr_2_O_3_@MnO_2-x_. For comparison, the MnO_2_-SS electrode, without chromium adsorption, was also calcined under the same conditions and named as MnO_2-x_.

### Electrochemical Test

The as-prepared electrodes were cut into a 14 mm discs and used as the cathode in non-aqueous lithium-oxygen batteries. The battery performance was tested in a lithium-oxygen battery developed in-house, with a lithium metal anode, a separator (GF/C, Whatman), 200 μl 1.0 M Lithium bis(trifluoromethanesulphonyl)imide (LiTFSI)-tetraethylene glycol dimethyl ether (TEGDME) electrolyte, and a cathode. The battery was assembled in a glove box and then tested in an O_2_ atmosphere, at a pressure of 1.25 atm.

The electrochemical performance of the electrode was first tested through electrochemical impedance spectra (EIS) and cyclic voltammetry (CV). All the tests were carried out in an electrochemical workstation (CHI660, Shanghai Chenhua). The discharge-charge tests were conducted on a battery testing system (CT-4008, Neware) at current densities of 200, 400, and 800 mA·g^−1^. The cycle stability was tested in a homemade Li^+^-oxygen batteries at the current density of 400 mA·g^−1^, with a fixed specific capacity of 1,000 mAh·g^−1^. A LiFePO_4_ electrode was used as the reference electrode and counter electrode in the Li^+^-oxygen batteries and the electrode was prepared as follows: lithium iron phosphate (LiFePO_4_, MTI Corporation), acetylene Black (AB, Canrd) and Poly tetra fluoroethylene (PTFE, Canrd) (85:5:10 wt%) were thoroughly mixed and pressed on a stainless steel mesh (16 mm diameter) and then dried under vacuum at 120°C for 12 h. To ensure the voltage stability, a ∼10-fold excess of LiFePO_4_ was applied in the Li^+^-oxygen batteries. All tests were carried out at room temperature (25 ± 1°C).

### Material Characterizations

The crystal structures of different samples were tested with an X-ray diffraction system (XRD, D/max2500/PC). The morphologies were obtained by scanning electron microscopy (SEM, S-4700) and scanning transmission electron microscopy (STEM, FEI TECNAI G^2^F20S-TWIN). The valence states of Mn and Cr were characterized by X-ray photoelectron spectroscopy (XPS) on an Axis Ultra spectrometer. The composition of the as-prepared manganese oxide was tested by the iodometry method. The discharge product was analyzed by XPS and Raman spectroscopy (RENISHAW in Via, wave length 532 nm). The morphology of the product was observed by SEM.

## Results and Discussion

### Material Characterizations

**FIGURE 1:**
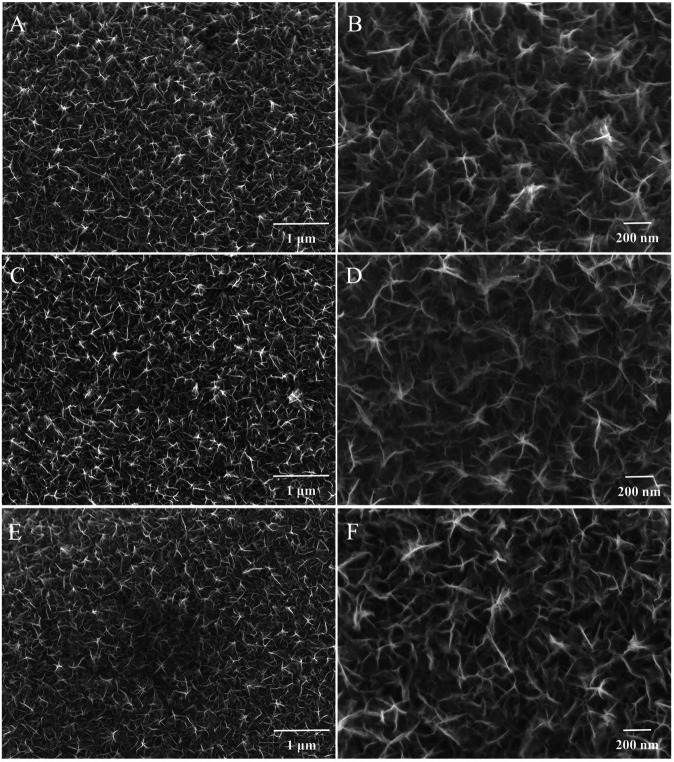
SEM images of **(A,B)** MnO_2_; **(C,D)** MnO_2-x_; and **(E,F)** Cr_2_O_3_/MnO_2-x_.

The SEM images of different electrodes were shown in Figure 1. From these images, we can clearly find that the MnO_2_ prepared by electrodeposition exhibits an irregular nanosheet structure, with a thickness of ∼50 nm. These nanosheets are expected to provide a high surface area for chromium adsorption and electrochemical reactions in lithium-oxygen batteries. After the simple calcination or adsorption-calcination process, the electrode morphology was characterized, as shown in Figures 1C–F, respectively. The results show that after the calcination or adsorption-calcination process, the surface morphology of the electrode remains unchanged. Therefore, the improvement in the charge-discharge performance of the electrode can be attributed to the change of the surface state.

**FIGURE 2:**
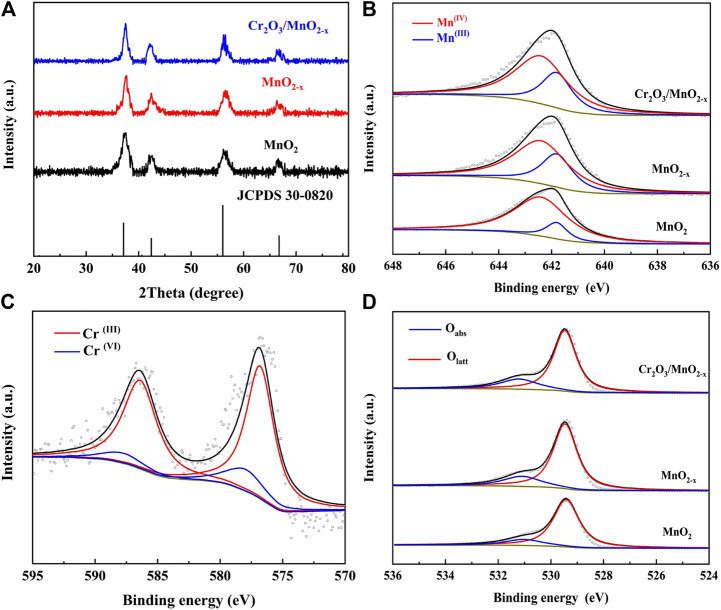
**(A)** XRD patterns for different electrode materials; **(B)** Deconvoluted Mn 2p3/2 spectra of different electrode materials; **(C)** Deconvoluted Cr (2p) spectra of Cr_2_O_3_/MnO_2-x_; **(D)** Deconvoluted O (1s) spectra of different electrode materials.

XRD and XPS were measured to investigate the composition and valence state of the elements of the different electrodes, as shown in Figure 2. To avoid the influence of the SS substrate, before the test, the electrode materials on the electrode surface were peeled away by ultrasonication and collected. XRD showed that these patterns exhibited a group of diffraction peaks at 37.1°, 42.4°, 56.0°, and 66.7°, which matched well with the diffraction of the (100), (101), (102), and (110) planes of the ε-MnO_2_ (akhtenskite, Joint Committee on Powder Diffraction Standards no. 33-0820). This result indicates that the as-prepared MnO_2_ was mainly ε-MnO_2_ and that after the adsorption-calcination process, the crystal structure did not change significantly.

XPS was used to identify the valence states of Mn, Cr, and O their species in the different samples. Figure 2B shows the deconvoluted Mn 2p_3/2_ peak of the different electrodes, where the peaks at 642.4 and 641.8 eV correspond to Mn (IV) and Mn (III), respectively. The results show that after the calcination/adsorption-calcination, the peak intensity of Mn (III) increased while the peak intensity of Mn (IV) decreased, revealing that the oxygen vacancies and the associated Mn (III) were generated during the heat treatment. These oxygen vacancies are expected to increase the catalytic oxygen reduction activity of the MnO_2_-based materials, thereby achieving high discharge capacity.

The existence of Cr on MnO_2-x_ nanosheets was studied by XPS, as shown in Figure 2C. The test results show that Cr mainly exists in the mixed form of Cr (III) and Cr (VI):The peaks located at 576.8 and 586.4 eV are corresponding to Cr (III) and the peaks located at 578 and 587.8 eV can be attributed to the Cr (VI) within the Cr_2_O_3_ (Yao et al., 2014; Gan et al., 2016). The mixed states of Cr in Cr_2_O_3_/MnO_2-x_ are expected to have high catalytic activity in promoting charge process. The O 1s spectrum, in Figure 2D, shows two peaks at 529.8, and 531.5 eV, which are correlated to the normal lattice oxygen (O_latt_), and adsorbed oxygen (O_abs_), respectively. Since the surface adsorbed oxygen mainly occurs at the oxygen vacancy, the oxygen vacancy content can be known through the analysis of O_abs_ (Li et al., 2020b; Mo et al., 2020). Through the O 1s spectrum, it can be found that the proportion of O_abs_ increases after the calcination and adsorption-calcination process, indicating that the process can introduce more oxygen vacancies in the MnO_2_ structure. The increase in oxygen vacancies is consistent with the Mn 2p spectrum and is expected to be beneficial for ORR. Iodometry and Inductively Coupled Plasma Optical Emission Spectrometer (ICP-OES) were also used to determine the composition of the different electrode materials. The composition of MnO_2_, MnO_2-x_ and Cr_2_O_3_/MnO_2-x_ was found to be MnO_1.99_, MnO_1.96_, and 0.004Cr_2_O_3_/MnO_1.96_, respectively.

**FIGURE 3:**
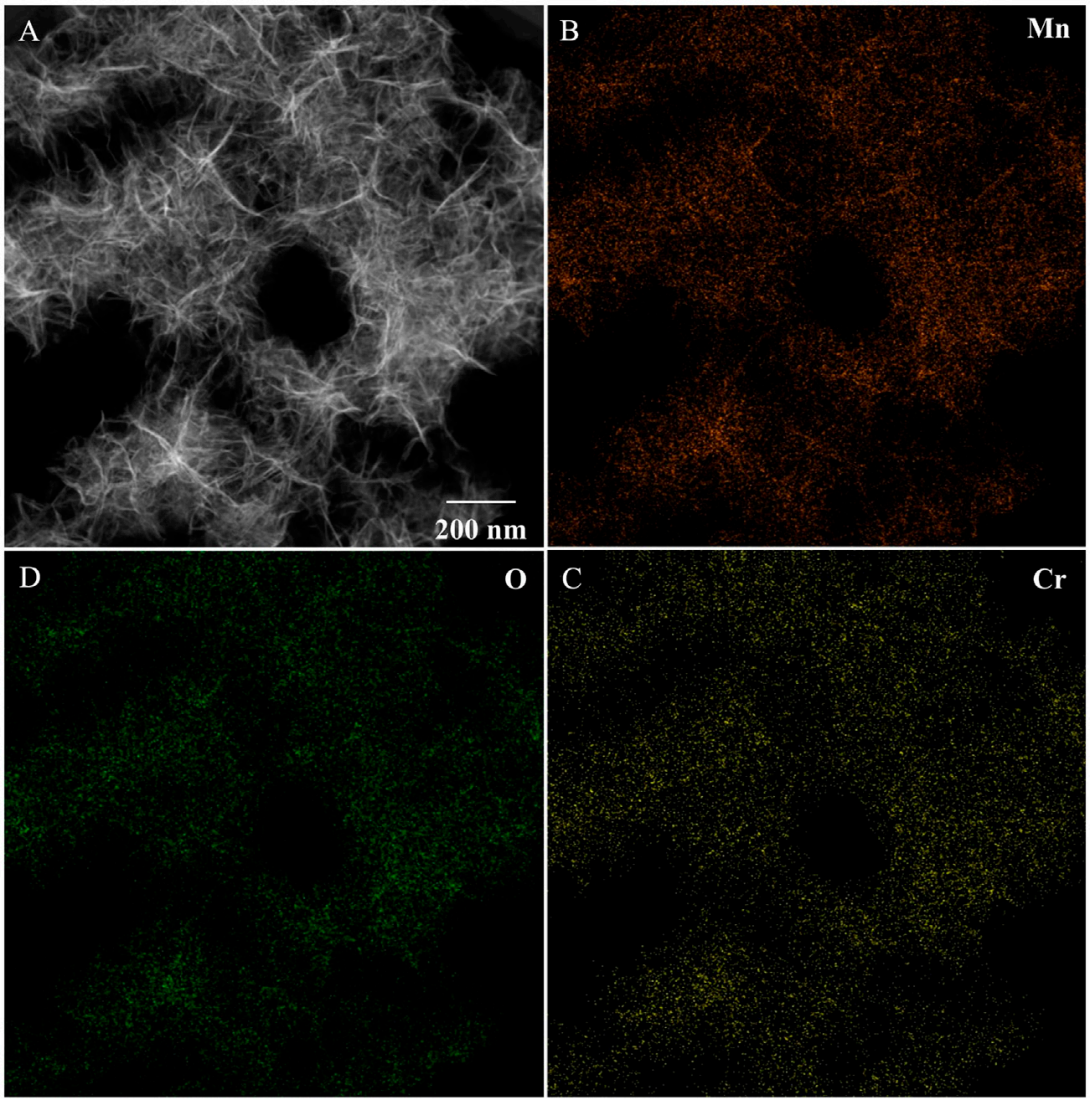
STEM **(A)** and EDS-mapping **(B–D)** images of Cr_2_O_3_/MnO_2-x_.

To investigate the distribution of Cr_2_O_3_ on the MnO_2-x_ nanosheets after adsorption-calcination, STEM-EDS mapping was carried out, as shown in Figure 3. The TEM images (Figure 3A) show that the electrodeposited MnO_2-x_ material is in the form of nanosheets, and the diameter of the Cr_2_O_3_/MnO_2-x_ nanosheet is consistent with that obtained from the SEM results. EDS mapping (Figures 3B–D) shows that chromium is evenly distributed on the MnO_2-x_ nanosheets. The surface Cr/Mn ratio of the Cr_2_O_3_/MnO_2-x_ was calculated to be 0.153 from the elements abundance test from STEM-EDS mapping (Supplementary Figure S1). This uniform dispersion can be attributed to the uniform adsorption process on the MnO_2_ surface. Thus, chromium can be uniformly loaded on the surface of MnO_2-x_ under low loading, which has two advantages: 1) the low Cr_2_O_3_ loading can minimize the inhibition effect to MnO_2-x_ for the discharge process of lithium-oxygen batteries; and 2) uniform chromium distribution is conducive to achieve uniform contact with the discharge product, Li_2_O_2_, so as to achieve high catalytic effect in the charging process.

From the SEM, XRD, XPS, iodometry and ICP-OES and TEM results, it can be concluded that: 1) a MnO_2_ film can be grown on the surface of the substrate by electrodeposition; 2) the adsorption process can adsorb chromium species on the surface of the MnO_2_; and 3) the subsequent calcination process introduces a large amount of oxygen vacancies and Mn (III) into manganese dioxide, and at the same time, converts chromium species to Cr_2_O_3_. The above results indicate that the Cr_2_O_3_ decorated MnO_2-x_ nanosheet electrode can be prepared by the combined electrodeposition-adsorption-calcination method. The electrode has a high oxygen vacancy content, low Cr_2_O_3_ loading, and uniform Cr_2_O_3_ distribution, and can deliver high discharge capacity and low charge voltage.

### Electrochemical Performance

**FIGURE 4:**
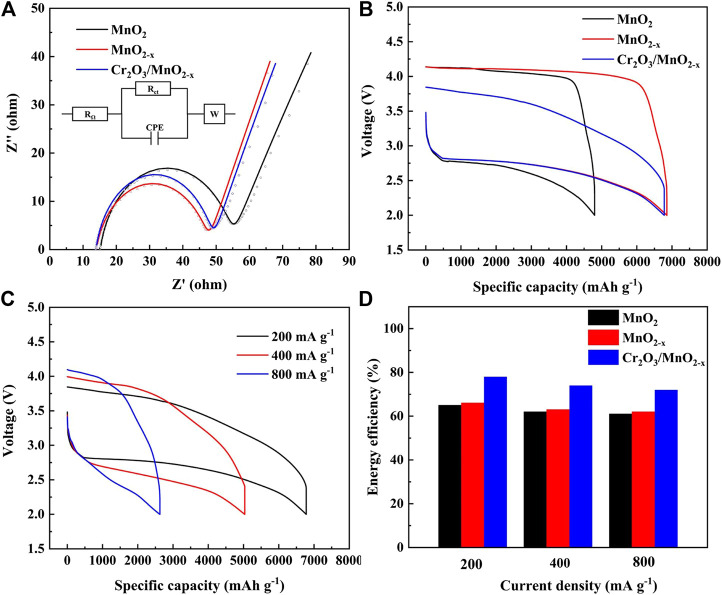
**(A)** EIS plots and simulated results for different electrode (insert: the equivalent circuit model for EIS); **(B)** Discharge-charge performances of different electrodes at 200 mA·g^−1^; **(C)** High current discharge-charge performances of the Cr_2_O_3_/MnO_2-x_ electrode; **(D)** Calculated energy efficiency of different electrodes at different current densities.

Before the charge and discharge test, the impedance characteristics of different electrodes in lithium-oxygen batteries were studied by AC impedance and fitted with a simple equivalent circuit mode, and the results are shown in Figure 4A. In this model, R_Ω_ corresponds to the ohmic resistance of electrolyte and electrode materials, R_ct_ represents charge transfer resistance for ORR/OER on the cathode-electrolyte interface, CPE is the constant phase element corresponds to the cathode-electrolyte interfaces and W is the finite length Warburg contribution. The fitting results show that the ohmic resistance of MnO_2_, MnO_2-x_ and Cr_2_O_3_/MnO_2-x_ electrode was calculated to be 15.1, 13.7 and 13.9 Ω, respectively. At the same time, the charge transport resistance of MnO_2_, MnO_2-x_ and Cr_2_O_3_/MnO_2-x_ electrode was calculated to be 43.4, 36.6 and 38.6 Ω, respectively. This result can be attributed to three reasons: 1) oxygen defects and Mn (III) generated during the calcination and adsorption-calcination process can increase the electronic conductivity of the MnO_2-x_ nanosheets, thereby reducing the ohmic resistance of the electrode material; 2) oxygen vacancies can improve the adsorption of oxygen species on the surface of the MnO_2_-based material, thereby obtaining good catalytic activity for oxygen reduction and lower charge transfer resistance; 3) the small amount of Cr_2_O_3_ is uniformly distributed on the MnO_2-x_ nanosheets, thereby reducing the inhibitory effect of Cr_2_O_3_ on oxygen reduction to a minimum. Therefore, the charge transfer impedances of the MnO_2-x_ electrode and the Cr_2_O_3_/MnO_2-x_ electrode are close in value.

Figure 4B shows the galvanostatic discharge/charge performance of the three electrodes in non-aqueous lithium-oxygen batteries, under a current density of 200 mA·g^−1^. The MnO_2_ electrode delivers a discharge capacity of 4,801 mAh·g^−1^ with a terminal charge voltage of 4.14 V. Under the same conditions, the MnO_2-x_ electrode and Cr_2_O_3_/MnO_2-x_ electrode exhibit specific discharge capacities of 6,854 mAh·g^−1^ and 6,779 mAh·g^−1^, respectively. More importantly, the Cr_2_O_3_/MnO_2-x_ electrode delivers a reduced terminal charging voltage of 3.84 V. The energy efficiencies of the MnO_2_, MnO_2-x_, and Cr_2_O_3_/MnO_2-x_ electrodes were calculated to be 66%, 65%, and 78%, respectively. This result suggests that the Cr_2_O_3_/MnO_2-x_ electrode exhibits a high specific capacity, the lowest charge voltage, and the highest energy efficiency.

MnO_2_-based materials can work as anode active materials in lithium-ion batteries through the lithiation and delithiation reactions, thus showing discharge-charge capacity. In order to exclude the capacity of lithiation and delithiation capacity, the specific capacities of different electrodes were tested in sealed button cells under the same current density. The results (Supplementary Figure S2) show that the lithiation and delithiation capacities of the MnO_2_, MnO_2-x_ and Cr_2_O_3_/MnO_2-x_ electrodes were about 225–250 mAh·g^−1^, which can be ignored compared with the capacity in lithium-oxygen batteries.

The rate capability of the as-prepared MnO_2_, MnO_2-x_ and Cr_2_O_3_/MnO_2-x_ electrodes are tested under different current densities, and the results are shown in Supplementary Figure S3, and Figures 4C,D, respectively. The Cr_2_O_3_/MnO_2-x_ electrode delivers specific discharge capacities of 6,779, 5,033, and 2,627 mAh·g^−1^ with the energy efficiencies 78%, 74%, and 72%, under the current densities 200, 400, 800 mA g^−1^, respectively (Figures 4C,D). At the same time, the MnO_2_ and MnO_2-x_ cathodes can deliver specific capacities of 4,801/6,854, 3,712/5,147, and 2,083/2,706 mAh· g^−1^ with energy efficiencies, 66/65%, 63/62%, and 62/61, respectively.

**FIGURE 5:**
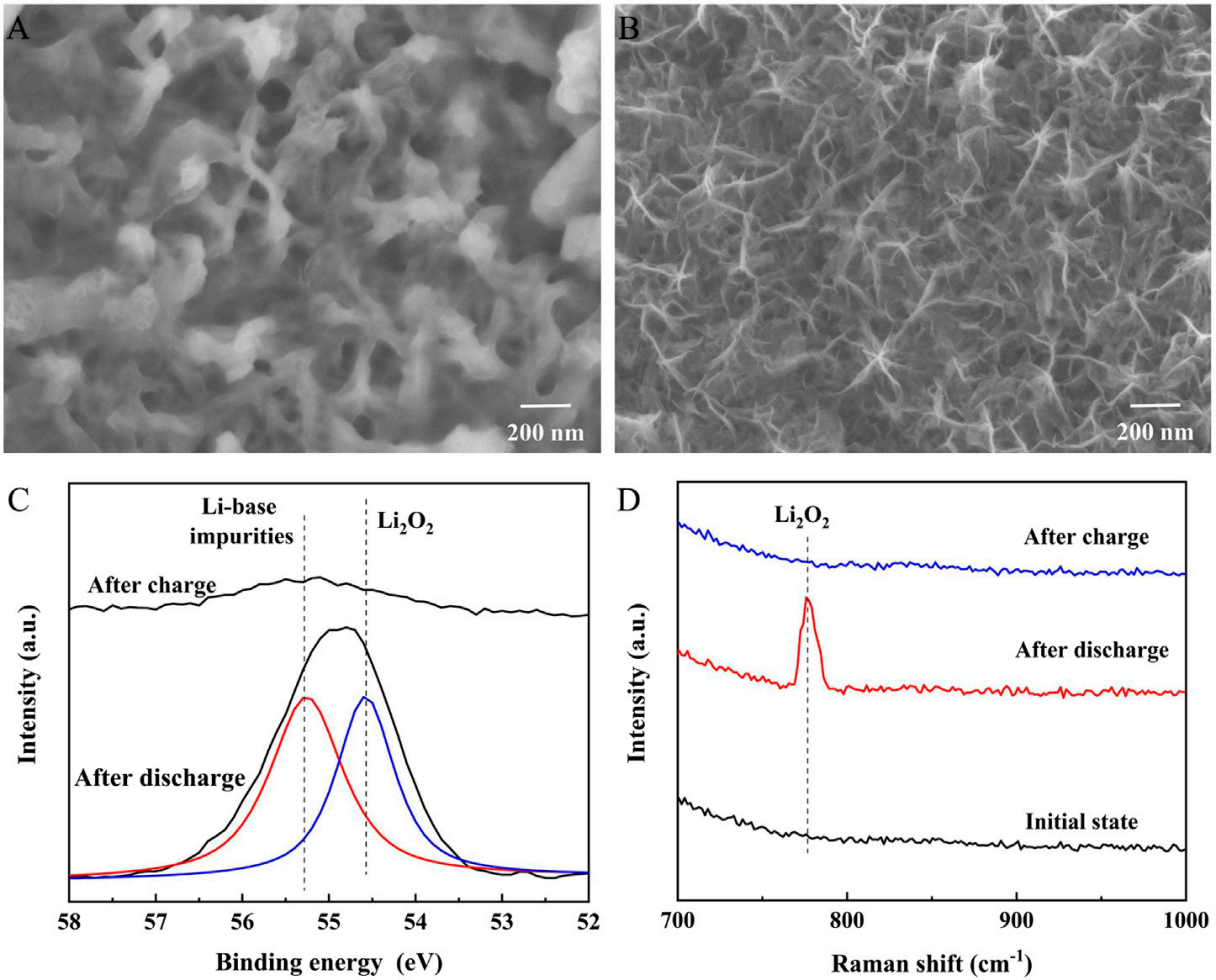
Characterizations of the discharge products on Cr_2_O_3_/MnO_2-x_: **(A)**, **(B)** morphology of the Cr_2_O_3_/MnO_2-x_ electrode after discharge and charge; **(C)** Li 1s spectra of the Cr_2_O_3_/MnO_2-x_ electrode after discharge and charge; **(D)** Raman spectra of the Cr_2_O_3_/MnO_2-x_ electrode after discharge and charge.

The discharge products of Cr_2_O_3_/MnO_2-x_ were characterized by SEM, XPS, and Raman spectroscopy, as shown in Figure 5. The SEM image (Figure 5A) shows that the surface of the nanosheets is covered by film-like products after discharge. This film-like product can achieve sufficient contact with the catalyst surface, ensuring good catalytic activity during charging. After charging, the SEM image (Figure 5B) shows that the film-like product completely disappears and that the electrode surface recovers to the same as before discharge. Li 1s XPS spectra (Figure 5C) were obtained from the electrode after cycling to analyze the composition of the discharge product. In the discharged electrode, the Li 1s peak was mainly composed of Li_2_O_2_ and a small amount of li-based impurities which come from contamination during testing or parasitic reactions, indicating that Li_2_O_2_ had decomposed on the catalyst during the charging process. Raman spectroscopy (Figure 5D) was also used to study the existence of discharge products on the electrode surface after cycling. The results show that the product after discharge was mainly in the form of Li_2_O_2_ and that the Li_2_O_2_ peak disappeared after charging, which is consistent with the XPS results.

**FIGURE 6:**
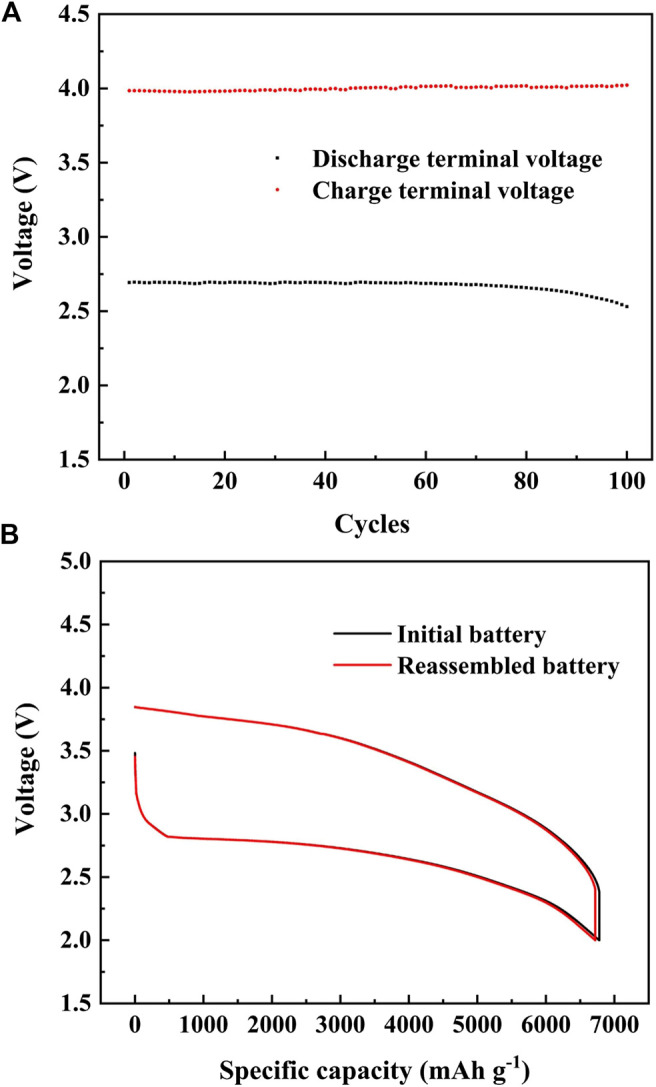
**(A)** Cycle performance of the Cr_2_O_3_/MnO_2-x_ cathode electrode under the current density of 400 mA·g^−1^ with the fixed capacity of 1,000 mAh·g^−1^; **(B)** Discharge-charge performance of the reassembled battery and the initial battery.

The stability of the Cr_2_O_3_/MnO_2-x_ electrode was tested in the in-house Li^+^-oxygen battery, using LiFePO_4_ as the counter electrode to eliminate the influence of lithium electrode on the cycle performance. The capacity of the battery was limited to 1,000 mAh·g^−1^, and the battery voltage and the cycle performance are shown in Figure 6A. This experimental result shows that the battery can stably cycle for 100 cycles in the voltage range of 2.0–4.5 V without capacity degradation, but its discharge terminal voltages decrease from 2.693 to 2.530 V with the charge terminal voltages increase from 3.985 to 4.022 V. This voltage change can be attribute to the accumulation of minor by-products rather than by material degradation. To verify this hypothesis, the cycled battery was disassembled, cleaned, and reassembled with a fresh lithium anode, separator, and electrolyte for testing. The discharge/charge performance in Figure 6B shows that the reassembled battery could achieve the same charge and discharge performance as that of a fresh battery, indicating good stability of the Cr_2_O_3_/MnO_2-x_ electrode.

## Conclusion

In this work, we have prepared an electrode based on Cr_2_O_3_ decorated MnO_2-x_ nanosheets as a non-carbon and binder-free cathode for lithium-oxygen batteries. The as-prepared Cr_2_O_3_/MnO_2-x_ electrode contains abundant oxygen vacancies and Mn (III) and uniformly distributed Cr_2_O_3_. With this novel electrode, a specific capacity of 6,779 mAh·g^−1^, terminal charge voltage of 3.84 V and energy efficiency of 78% were achieved in the non-aqueous lithium-oxygen battery. In addition, this electrode also showed good performance in rate capability tests. SEM, Raman spectroscopy, and XPS demonstrate that the film-like Li_2_O_2_ is deposited on the surface of the electrode as the main discharge product and is fully decomposed in the subsequent charging process. Furthermore, the cycling performances of freshly assembled and reassembled batteries show the good stability of the Cr_2_O_3_/MnO_2-x_ electrode. Thus, this work shows that the Cr_2_O_3_/MnO_2-x_ electrode is an important candidate for non-aqueous lithium-oxygen batteries.

## Data Availability

The original contributions presented in the study are included in the article/Supplementary Material, further inquiries can be directed to the corresponding authors.
