# Supplementary figures and images for: A Novel Cr2O3/MnO2-x Electrode for Lithium-Oxygen Batteries with Low Charge Voltage and High Energy Efficiency
Source: Front Chem. 2021 Feb 1;9:646218. doi: 10.3389/fchem.2021.646218 (PMC7958876; doi:10.3389/fchem.2021.646218)

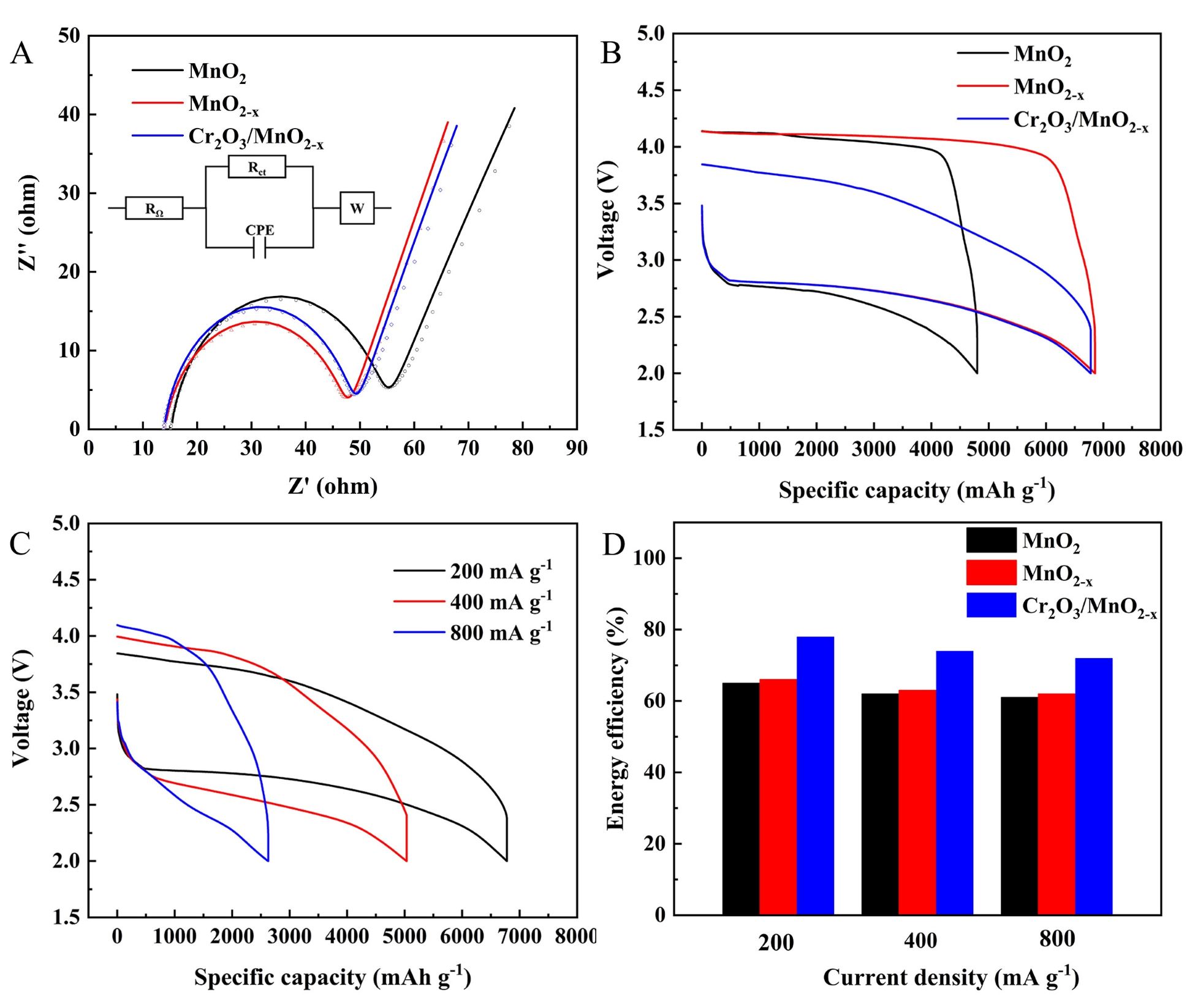

Supplement: Supplementary file 1 [file datasheet1.zip › ╠ß╜╗═╝╞1⁄4/═╝-4-REVISE.jpg]

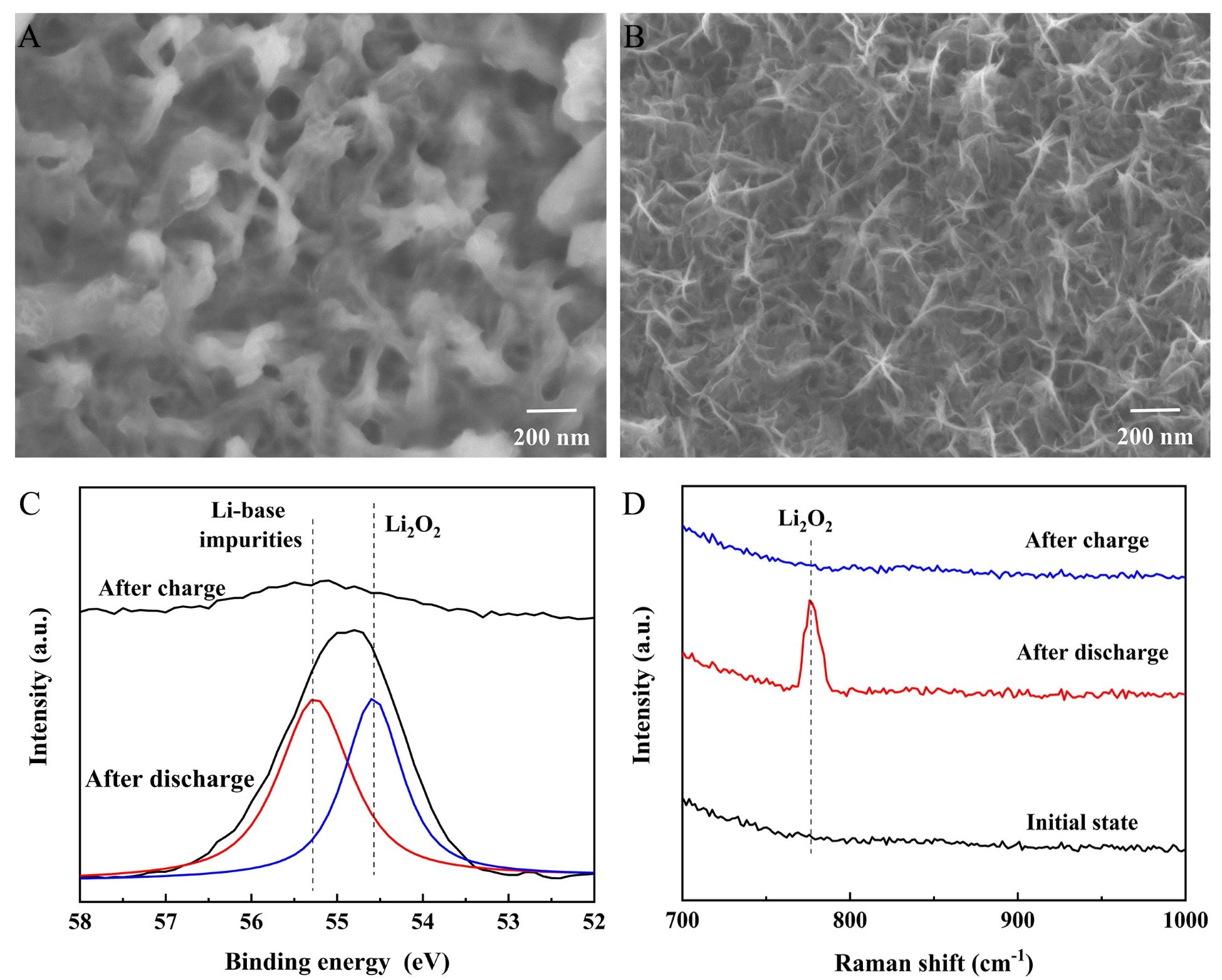

Supplement: Supplementary file 1 [file datasheet1.zip › ╠ß╜╗═╝╞1⁄4/═╝-5.jpg]

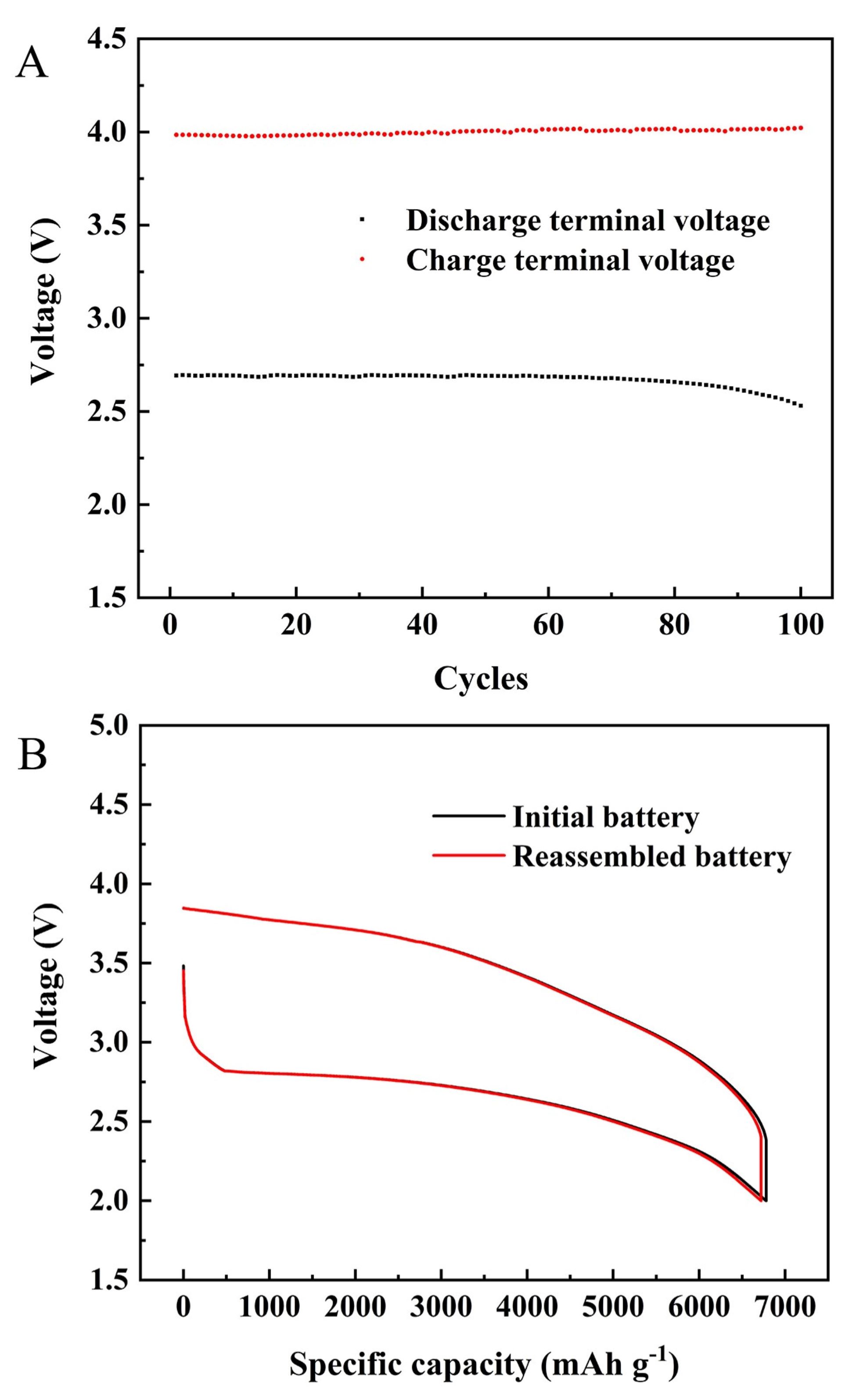

Supplement: Supplementary file 1 [file datasheet1.zip › ╠ß╜╗═╝╞1⁄4/═╝-6.jpg]

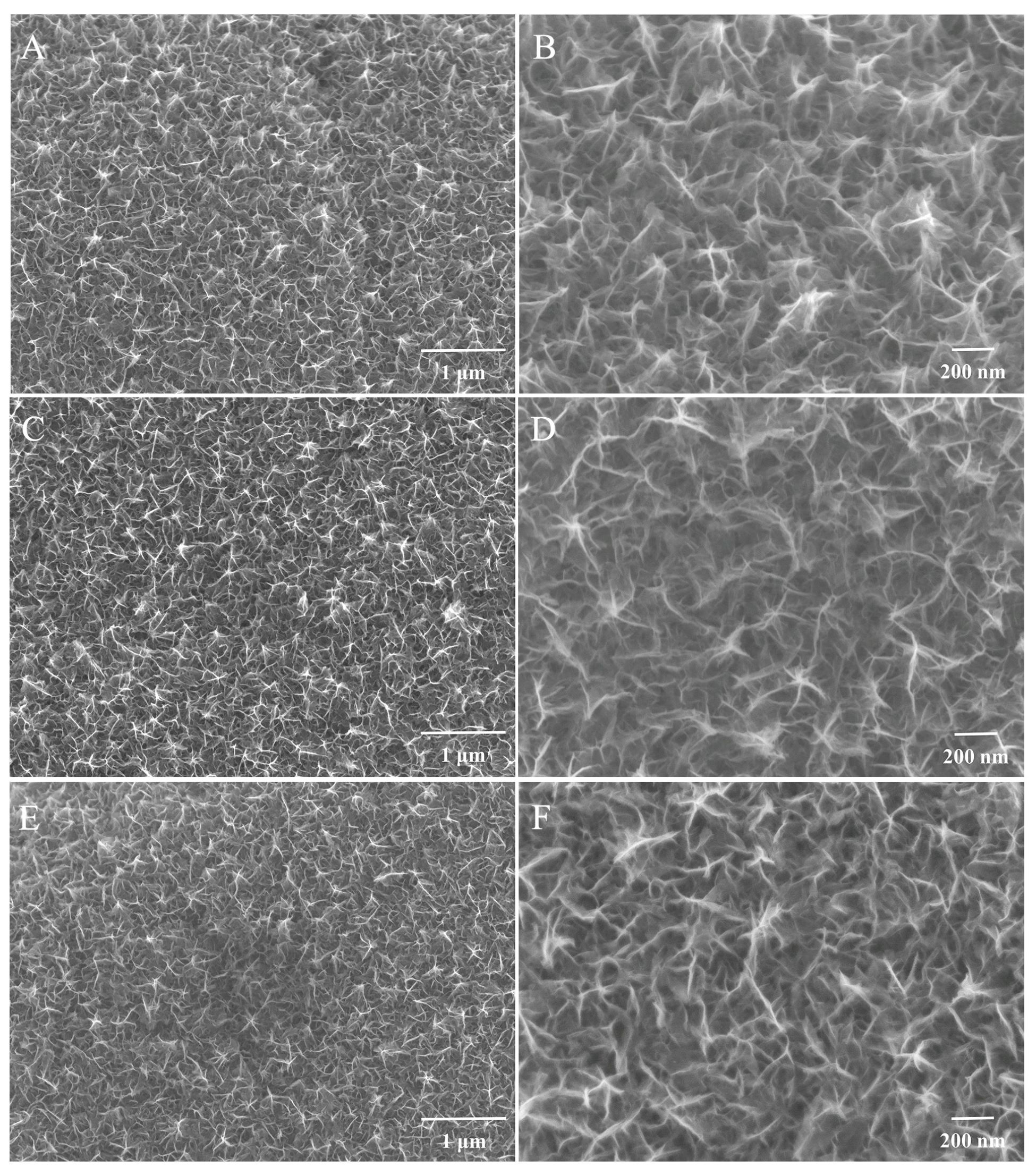

Supplement: Supplementary file 1 [file datasheet1.zip › ╠ß╜╗═╝╞1⁄4/═╝1.jpg]

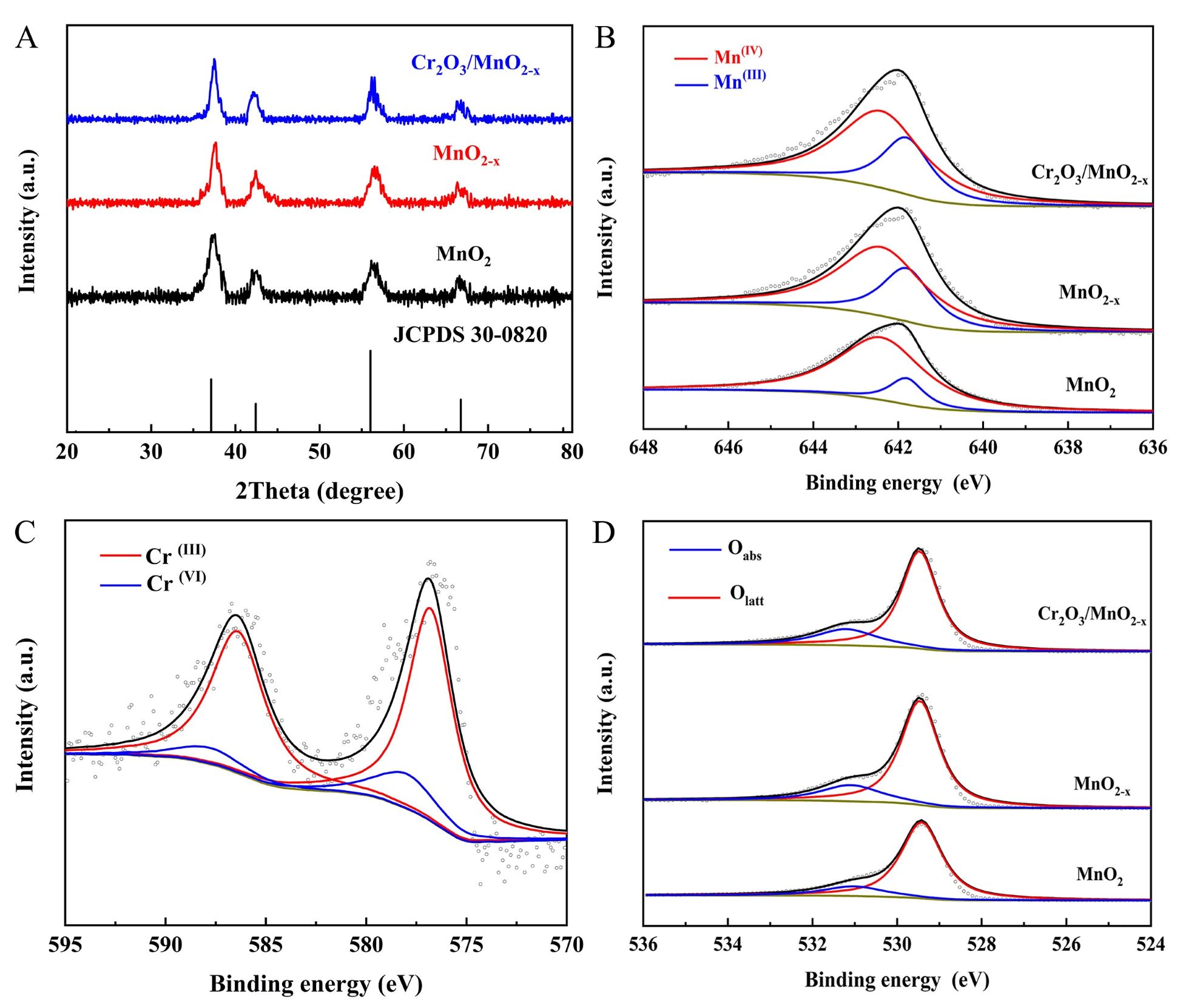

Supplement: Supplementary file 1 [file datasheet1.zip › ╠ß╜╗═╝╞1⁄4/═╝2.jpg]

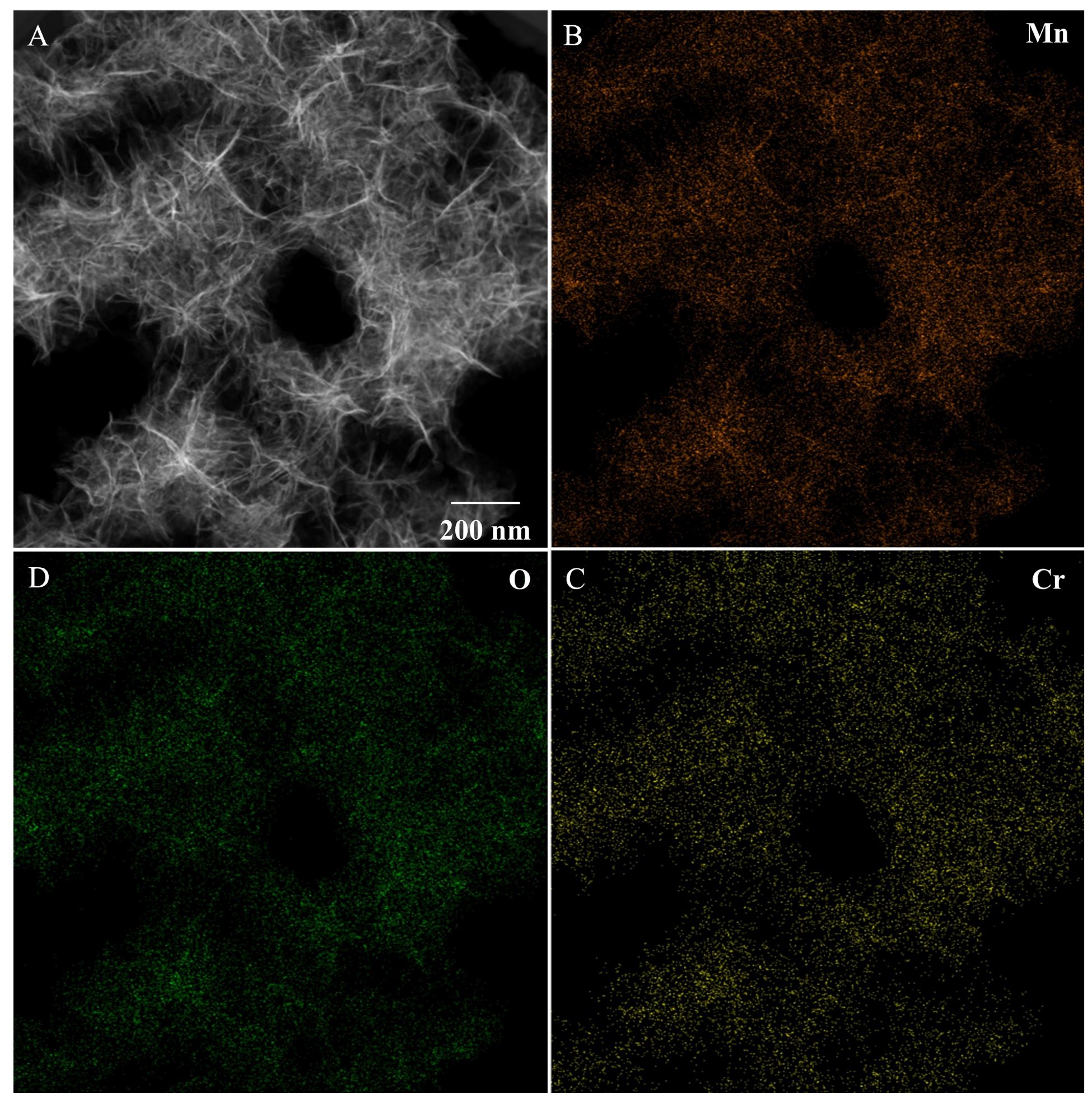

Supplement: Supplementary file 1 [file datasheet1.zip › ╠ß╜╗═╝╞1⁄4/═╝3.jpg]
