# Supplementary material for: A Novel Cr2O3/MnO2-x Electrode for Lithium-Oxygen Batteries with Low Charge Voltage and High Energy Efficiency
Source: Front Chem. 2021 Feb 1;9:646218. doi: 10.3389/fchem.2021.646218 (PMC7958876; doi:10.3389/fchem.2021.646218)
Supplement: Supplementary file 2 [file table1.docx]

**A Novel Cr_2_O_3_/MnO_2-x_ Electrode for Lithium-oxygen Batteries with Low Charge Voltage and High Energy Efficiency**

**Zhaohuan Wei^1^*, Zhiyuan Zhang^1^, Yaqi Ren^2^*, Hong Zhao^3^**

^1^School of Physics, University of Electronic Science and Technology of China, Chengdu, China.

^2^School of Materials and Environmental Engineering, Chengdu Technological University, Chengdu, China.

^3^School of Materials Science and Energy Engineering, Foshan University, Foshan, China.

*** Correspondence:**Dr Zhaohuan Wei and Yaqi Ren
**zhwei@uestc.edu.cn**，**renyaqii@163.com**

**
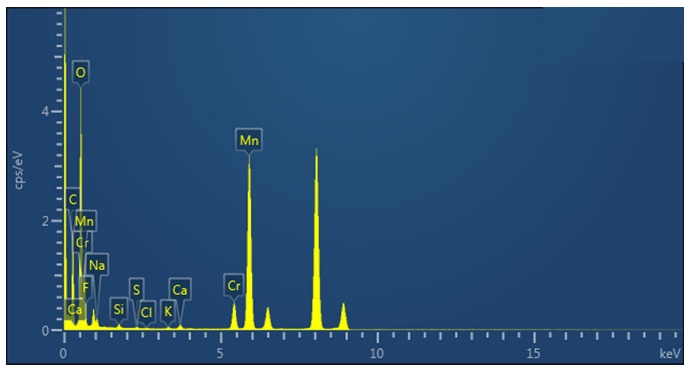
**

Figure S1 Abundance of different elements on the Cr_2_O_3_/MnO_2-x_ electrode surface


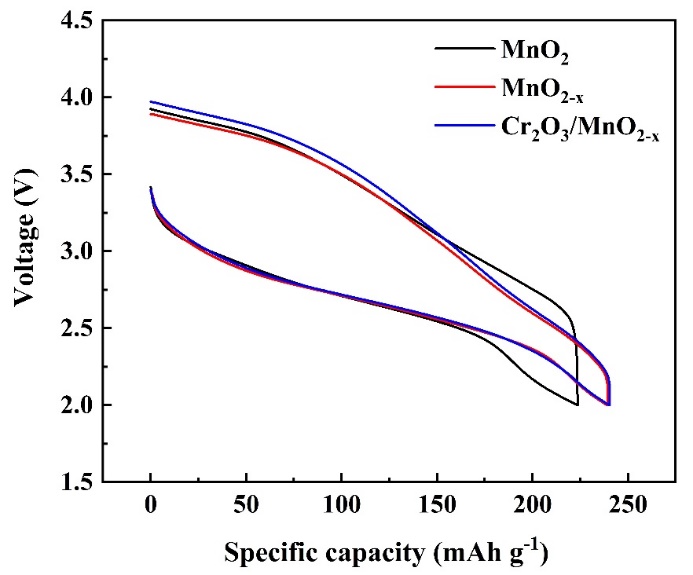


Fig. S2 Discharge-charge performances of different electrodes in Ar.

**
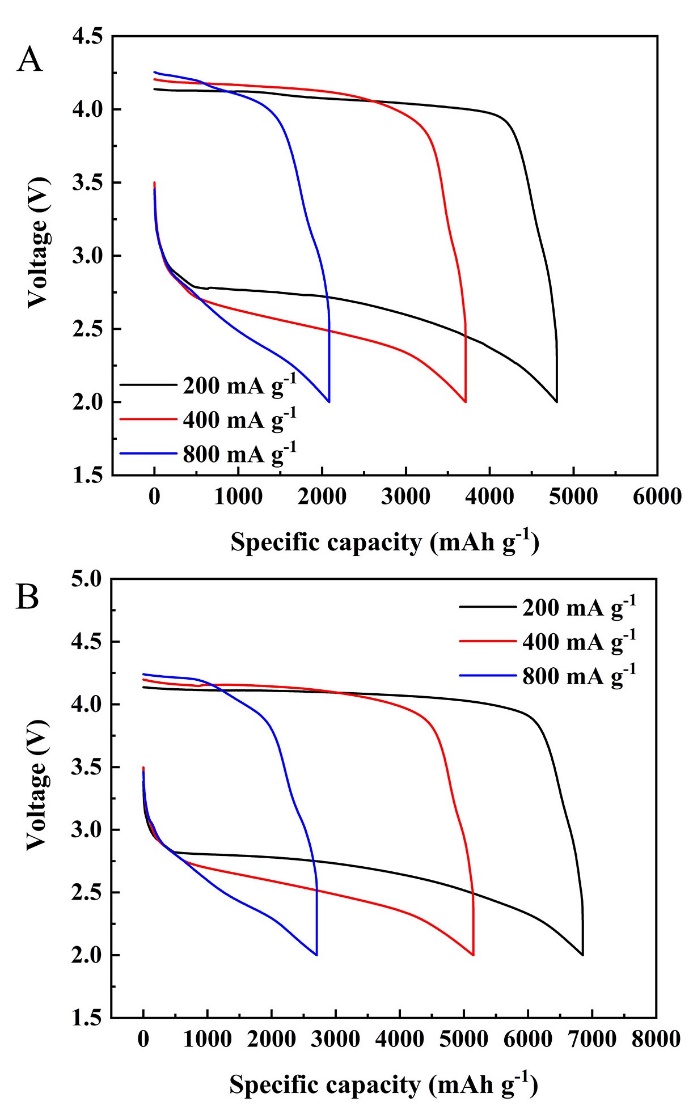
**

Fig. S3 High current discharge-charge performances of the (A) MnO_2_ electrode and (B) MnO_2-x_ electrode
